# Editorial: Studying Tree Responses to Extreme Events

**DOI:** 10.3389/fpls.2017.00506

**Published:** 2017-04-20

**Authors:** Achim Bräuning, Andreas Bolte, Cristina Nabais, Sergio Rossi, Ute Sass-Klaassen

**Affiliations:** ^1^Department of Geography and Geosciences, Institute of Geography, Friedrich-Alexander-University Erlangen-NurembergErlangen, Germany; ^2^Thünen Institute of Forest EcosystemsEberswalde, Germany; ^3^Departamento de Ciências da Vida, Faculdade de Ciências e Tecnologia, Centro de Ecologia Funcional, Universidade de CoimbraCoimbra, Portugal; ^4^Département des Sciences Fondamentales, Université du Québec à ChicoutimiChicoutimi, QC, Canada; ^5^Key Laboratory of Vegetation Restoration and Management of Degraded Ecosystems, Provincial Key Laboratory of Applied Botany, South China Botanical Garden, Chinese Academy of SciencesGuangzhou, China; ^6^Forest Ecology and Forest Management Group, Wageningen University and ResearchWageningen, Netherlands

**Keywords:** climate change, future forests, tree, mechanistic understanding, structure-function relationships, long-term monitoring, intra-annual resolution, resilience

## Climate change and tree response to extreme events

There is a common understanding that climate change is a global challenge in the twenty-first century for the future of humankind (Stott et al., 2016). It is meanwhile clear that human activities have influenced the earth climate system, with substantial modifications in the frequency and magnitude of climate extreme events that occurred since the 1950s (IPCC, 2013, AR5).

Among climate extreme events, hydric as well as thermal anomalies such as droughts, flooding, heat waves, fires, and frost events play an important role for productivity and survival of trees and may cause severe disturbances in forest ecosystems (Allen et al., 2010; Teskey et al., 2015). Trees are long-living organisms with a life-span of between several hundred to thousands of years, with the oldest living tree ramets on earth having reached ages of up to 5,000 years (Stahle, 1996/1997). Thus, mature forest ecosystems may persist for many decades or centuries without considerable variation in tree species composition, also due to cyclic regeneration processes (Zukrigl et al., 1963; Fischer, 1997; Körner, 2013). Disturbances, however, may induce abrupt changes of ecosystem structure and species composition, leading to multiple and less predictable successional pathways (Swanson et al., 2011). The long-term structural persistence of forests strongly depends on the adaptive capacity/plasticity of the species, resulting from both tolerance and resilience potential of tree *individuals* to environmental impacts, e.g., due to climate extreme events.

These arguments inspired the present research topic, which mostly involves papers on a tree-centered approach that explicitly addresses the adaptive capacity of trees at individual, sub-species, and species levels. With this, a reliable basis shall be provided for shaping and managing adaptive, climate-resilient future forests, or to restore landscapes with tree species more suitable or adapted to future environmental conditions (Millar et al., 2007; Bolte et al., 2009; Jacobs et al., 2015).

The papers presented in this research topic derived from the activities conducted during 2012 to 2016 in the EU COST Action FP1106 STReESS (Studying Tree Responses to extreme Events: a SynthesiS). The STReESS community consisted of more than 150 scientists from 34, mostly European, countries, active in the fields of wood anatomy, dendrochronology, ecophysiology, tree modeling, forest ecology, forest management, and forest genetics. This COST Action addressed and answered questions on the impact of extreme climate events on forests and trees, with a special focus on drought.

### Tree-centered perspective

The STReESS research methodology was based on a bottom-up tree-centered perspective. A tree-centered approach has demonstrated to be suitable to assess impacts of extreme climatic events on trees and forest stands at different spatio-temporal resolutions, and to predict how tree species at scales ranging between the individual to the population might adapt or respond to different future climate conditions (Sass-Klaassen et al.). This framework (i) provides a basic understanding of how climate extremes affect forests in terms of individual mortality, regeneration, and physiological adaptation; (ii) forms an ideal scale to study impacts of climate on morphological, wood-anatomical, and physiological responses of trees to their changing environment; and (iii) increases the perception of the legacy of climatic extremes that are imprinted in wood-anatomical structures that may affect the tree's ability to respond to future climate conditions, leading to possible aggravations of effects in case of repeated events of similar magnitude.

The COST Action was divided into specific topics dedicated to relevant aspects of tree response to climate extremes. Accordingly, the 40 papers included in this research topic can be grouped into the topical groups briefly summarized in the following sections.

#### Physiological adaptive traits

Extreme events, such as drought, heatwaves, flooding, or frost, affect trees via impacting vital physiological processes, such as water transport (Oberhuber et al.), carbon assimilation, carbon mobilization (Beikircher et al.), or carbon concentration (Lintunen et al.).

Physiological traits associated with water transport and drought susceptibility include specific hydraulic conductivity (Rita et al.), vulnerability to cavitation (P50 value) (David–Schwartz et al.; López et al.; Rosner et al.), stomatal conductance (Seidel et al.), and sapflow (Steppe et al., 2015). All these physiological traits closely interact with wood-anatomical characteristics which vary between and within species, but also within individuals across time (Rathgeber et al.).

These changes in functional xylem (and phloem; Gričar et al.) anatomical traits in time clearly reflect phenotypic plasticity and define the long-term adaptive potential of physiological and wood-anatomical traits (Sass-Klaassen et al.). This is important as the plasticity of certain traits can increase the resilience of trees to climate warming (Sterck et al.).

#### Assessing frequency and impact of extreme events

An indispensable requirement for assessing the frequency of extreme climate events but also the long-term impact of climate factors on trees is the availability of long-term records. The cascading effect of extreme events on physiological and growth responses leads to distinct traces in the wood that hence can serve as biological proxies. The tree-centered approach uses long continuous tree-ring width records as well as discrete records of specific wood-anatomical features in the tree ring to precisely reconstruct past climate conditions and to exactly date extreme climate events (Carrer et al.; Kurz-Besson et al.). Specific tree-ring features such as intra-annual density variations (Zalloni et al.; Klisz et al.), flood rings (Copini et al.), or the absence of tree rings in specific years (Novak et al.) are reliable proxies (“markers”) of drought, floods, or other extreme events. Advances in knowledge on the intra-annual dynamics of wood formation provide information on the species-specific timing of growth in relation to climate (Martinez Del Castillo et al.) or under particular site conditions (Gričar et al.) and allows to increase the temporal resolution of intra-annual density variations (De Micco et al.) or flood rings (Copini et al.). Moreover, intra-annual measurements allow assessing directly the impact of the environment and its changes (Dao et al.; Maeght et al.; Legave et al.; Lechthaler et al.) or related effects such as defoliation (Guada et al.) on tree viability and growth dynamics. Within STReESS, big efforts have been made to verify the causal relationship between extreme climate events and wood-anatomical markers, including the aspect of temporal precision of markers. Databases and catalogs on specific wood-anatomical markers such as intra-annual density variations (IADFs) have been generated with the potential to study temporal and spatial patterns in extreme events for various mostly Mediterranean conifer species (Zalloni et al.).

#### New methods and tools to measure and evaluate stress markers in trees

Through collaboration between wood anatomists, ecophysiologists and tree modelers, huge advances have been made in developing and presenting new methods and tools to assess the mechanisms of tree responses to extreme climate events, but also to enable creation of long-term time series of cell-based wood-anatomical traits with reasonable time effort. Rathgeber et al., von Arx et al., von Arx et al., and Anfodillo et al. shed light on basic aspects of xylem-cell formation, quantification of wood-anatomical features, and important considerations for their interpretation. Other authors introduce new statistical tools for the evaluation of extreme climate events (Siegmund et al.) and highlight gaps in the understanding of the formation of specific wood-anatomical markers (Battipaglia et al.). The combination of high-time resolution monitoring of physiological processes (sapflow) and tree growth (dendrometers) forms a powerful approach to parameterise process-based tree models in real-time (Steppe et al.). Besides forming the bases for increased mechanistic understanding of tree responses to extreme events TreeWatch.net will improve the public awareness for climate-impact research on trees.

While yet only few trees are fully equipped with sophisticated monitoring tools, like TreeWatch.net, there is ample information on high-resolution tree growth available from thousands of dendrometers installed on numerous tree species in forests across the world. Firsts steps for collection and homogenisation of these measurements have been done and new analyses methods have been developed (Van der Maaten et al., 2016).

#### Varying adaptive capacity of populations to drought—facilitation of forest adaptation

One focus of the COST action was the analysis of inter-population adaptive capacity of trees to extreme events. Several studies addressed the adaptation to drought of pedunculate oak (Mijnsbrugge et al.; Turcsán et al.), European beech (Cocozza et al.; Bolte et al.; Hajek et al.), and Scots pine (Seidel and Menzel; Seidel et al.). Interestingly, a considerable variation in drought-stress tolerance or resilience among populations or provenances was observed. The results demonstrate the local adaptation of the studied tree species (Mijnsbrugge et al.; Seidel and Menzel,; Bolte et al.). However, the wide intra-population genetic variability influences the adaptive capacity of species and has to be also considered (Hajek et al.; Gershberg et al.). The outcomes strongly support the idea of the selection of drought-tolerant ecotypes or even individuals for increasing the adaptive capacity of forest stands with major European tree species within or even beyond their current native range (“assistant migration” sensu Millar et al., 2007). This includes the translocation of pre-adapted individuals and ecotypes, so called assisted gene flow (AGF), in order to facilitate the adaptation of planted stands or mixed stands with planted and natural regeneration (Aitken and Bemmels, 2016).

#### Practical relevance—consequences for forest management

The aim of COST STReESS was to test the potential of the multi-disciplinary tree-centered approach to assess short- and long-term effects of changing climate conditions and specifically extreme events on growth responses and thresholds for mortality (e.g., Cailleret et al., 2017). The advantage of such a bottom-up approach is that through enhanced mechanistic understanding, the plasticity of functional traits and hence the adaptive potential of populations and tree species under changing climate conditions can be estimated. This forms the bases for assessing the implications of changing climate conditions for the stability and productivity of different tree species. The actual challenge is linking the theory to the application, i.e., translating the progress that is made in ecophysiological and forest ecological research to recommendations for practitioners of forest management. Concrete examples are related to species and provenance selection. Based on the tree-centered approach, it is possible to develop and point out specific ecophysiological and wood-anatomical indicators for selecting drought-tolerant provenances or tree species (David-Schwartz et al.; Hajek et al.; Bolte et al.; Kurz-Besson et al.).

### The long term perspective

#### Achievements and future perspectives

The tree-centered approach has led to new insights on the impact of climate and extreme climate events on tree-growth processes, the structural components of wood, and the consequences for physiological performance of trees at individual to species level. The collection of articles in this research topic, together with more than 100 articles published elsewhere by the COST STReESS community on concepts (e.g., Steppe et al., 2015), meta-analysis of phenological wood traits (e.g., Rossi et al., 2013, 2016; Cuny et al., 2015), and important mechanisms behind mortality (e.g., Cailleret et al., 2017), illustrates the potential of this bottom-up approach. Major achievements include (1) enhanced understanding of relation between structure and function both on whole-tree level but specifically in woody tissues, (2) improvement of mechanistic models by parameterisation with high-time resolution measurements, (3) developing the basis for linking short-and long-term tree-growth and wood-anatomical records to assess long-term effects of extreme climate events on tree growth. Many aspects, processes, species, and traits have still to be studied in depth. Comprehensive mechanistic models linking structure, physiology, and function of tree species remain challenging, and require further multidisciplinary development of integrative conceptual and statistical approaches. The interdisciplinary network created through STReESS together with the effort that has been made on creating large databases and catalogs and advancing and harmonizing methods for data acquisition, data analyses, and modeling forms the bases for the necessary next steps.

#### Integration with other approaches

The findings of the COST Action STREeSS show evidence that the tree-based level provides opportunities to study cellular, genetic, physiological, anatomical, and ecological responses to climate as a whole. For example, several recent studies reported that seedlings of European beech from different climate origins over Europe performs differently in terms of drought tolerance (cf. Eilmann et al., 2014; Thiel et al., 2014; Pšidová et al., 2015; Dounavi et al., 2016). However, research activities within STREeSS showed that drought resistance is more related to local precipitation conditions at the place of origin than with geographically marginal origin (Bolte et al.). Hence, the role of local genetic variation in beech populations determining phenotypic plasticity in functional and structural traits of beech individuals that control drought adaptability need to be further evaluated (Cocozza et al.) to provide the most suitable plant material for forests adapted to future climates.

Another challenge remains the upscaling from tree-individual based drought responses to forest stands, since vegetation models are known to under-represent drought induced mortality (Steinkamp et al., 2015). Applying the tree-individual approach (Sass-Klaassen et al.) in systematically designed ways over species distribution ranges or ecological gradients in regional forested landscapes may have great potential to contribute to the debate of forest resilience to climate change (e.g., Reyer et al., 2015; Babst et al., 2017).

A third integrative pathways addresses the inclusion of high-resolution data on tree diameter growth and sapflow within the TreeWatchNet (Steppe et al.) in existing large-scale forest ecosystem monitoring networks like the UN-ECE ICP Forests on air pollutant and climatic impacts (Michel and Seidling, 2016) and the ICOS network on carbon flux monitoring (Laurent, 2016). High-resolution growth and physiology monitoring provides needed data to assess dynamic response of trees and forests to stressors and functioning in carbon and nutrient cycling.

## Author contributions

All coauthors acted as topic editors for the special issue in Frontiers in Plant Science entitled “Studying Tree Responses to extreme Events” All co-authors prepared the editorial text together and equally shared the tasks of writing, proofreading, and correcting the manuscript. ABr initiated the idea of the special issue and orgnaized the writing process of the editorial, with support from ABo and US.

## Funding

COST (European Cooperation in Science and Technology) is a funding agency for research and innovation networks. COST Actions help connect research initiatives across Europe and enable scientists to grow their ideas by sharing them with their peers. This boosts their research, career and innovation www.cost.eu.

### Conflict of interest statement

The authors declare that the research was conducted in the absence of any commercial or financial relationships that could be construed as a potential conflict of interest.
